# Role of proteolytic enzymes in human prostate bone metastasis formation: *in vivo* and *in vitro* studies

**DOI:** 10.1038/sj.bjc.6600207

**Published:** 2002-04-08

**Authors:** C A Hart, L J Scott, S Bagley, A A G Bryden, N W Clarke, S H Lang

**Affiliations:** Cancer Research UK - Group of Experimental Haematology, Paterson Institute for Cancer Research, Christie Hospital NHS Trust, Wilmslow Road, Manchester M20 4BX, UK; Department of Urology, Salford Royal Hospital, Eccles Old Road, Salford, Manchester, M6 8HD, UK; Christie Hospital NHS Trust, Wilmslow Road, Manchester M20 4BX, UK; YCR Cancer Research Unit, Biology Department, The University of York, York YO10 5YW, UK

**Keywords:** prostate cancer, prostate epithelia, bone marrow stroma, metastasis, urokinase plasminogen activator, matrix metalloproteinase

## Abstract

Prostate cancers ability to invade and grow in bone marrow stroma is thought to be due in part to degradative enzymes. The formation of prostate skeletal metastases have been reproduced *in vitro* by growing co-cultures of prostatic epithelial cells in bone marrow stroma. Expression of urokinase plasminogen activator, matrix metalloproteinase 1 and 7 by prostatic epithelial cells were identified using immunocytochemistry. Also, *in vivo* tissue sections from human prostatic bone marrow metastases were stained. To establish the role of these enzymes on colony formation, inhibitory antibodies directed against urokinase plasminogen activator, matrix metalloproteinase 1 and matrix metalloproteinase 7 were added into primary prostatic epithelial cells and bone marrow stroma co-cultures. All prostatic epithelial cell cultures stained positively for matrix metalloproteinase 1, matrix metalloproteinase 7 and urokinase plasminogen activator. Generally prostatic epithelial cells derived from malignant tissues showed increased staining in comparison to epithelia derived from non-malignant tissue. In agreement with *in vitro* co-cultures, the *in vivo* tissue sections of prostate bone marrow metastases showed positive staining for all three enzymes. Inhibition studies demonstrated that blocking matrix metalloproteinase 1, matrix metalloproteinase 7 and urokinase plasminogen activator function reduced the median epithelial colony area significantly in bone marrow stroma co-cultures *in vitro*. Using a human ex-vivo model we have shown that matrix metalloproteinase 1, matrix metalloproteinase 7 and urokinase plasminogen activator play an important role in the establishment of prostatic epithelial cells within bone marrow.

*British Journal of Cancer* (2002) **86**, 1136–1142. DOI: 10.1038/sj/bjc/6600207 www.bjcancer.com

© 2002 Cancer Research UK

## 

Prostate cancer (CaP) is the second commonest cancer in males in the UK with 9500 deaths in 1999 (CRC, 2001/2002) and commonly metastasises to the bone marrow (Jacobs, 1983). Fifty per cent of patients who present clinically with CaP already have incurable bone marrow metastases (George, 1988) and morbidity and death is usually a direct consequence of these. Therefore it is of importance that the mechanisms involved in the metastatic process be understood.

Tumour cells need to penetrate the extra-cellular matrix (ECM) and basement membrane in order to metastasise. The major components of the ECM are collagens (types I-V), proteoglycans, elastin, laminin and fibronectin, and in order to break free from the primary site and invade at the secondary site, tumour cells must overcome these structural barriers (Woolley, 1993). In recent years the interactions of various proteases have been identified in this crucial step, including the matrix metalloproteinase (MMP) and the serine protease family (plasmin and urokinase plasminogen activator (uPA)). The relative contributions of these proteases in tumour cell invasion has been in debate. More recent data have implicated the MMP family in a more complex role than ECM degradation alone. They have also been found to be actively involved with generating factors that can stimulate tumour cell (Stetler-Stevenson and Yu, 2001) and endothelial migration and angiogenesis needed for tumour formation (John and Tuszynski, 2001). The matrix metalloproteinase family comprises at least 20 zinc dependent endopeptidases clearly identified, with more (up to MMP-26) currently being characterised (Marchenko *et al*, 2001). Normally they are associated with programmed cellular events such as tissue remodelling, wound healing, uterine and breast involution and organogenesis in development (Chambers and Matrisian, 1997). Originally members of the MMP family were thought to be substrate specific. However, it has been recognised that each can degrade multiple substrates (John and Tuszynski, 2001).

In this study we looked at specific proteases that could be active in CaP and its secondary metastatic site, the bone marrow. MMP-7 (matrilysin) exhibits high activity against collagen IV, a major component of bone marrow stroma (BMS) and other basement membranes and has been found previously in carcinoma cells of many cancers including prostate and their metastases (Pajouh *et al*, 1991). In contrast, MMP-1 (interstitial collagenase) has a high substrate affinity for collagen I, also a major component of BMS (Takahashi *et al*, 1994). MMP-1 is thought to be expressed mainly by fibroblasts/stromal cells and is involved with interactions between the tumour cells and host fibroblasts in the remodelling of the ECM (Heppner *et al*, 1996). MMP-1 production has also been found to be up-regulated during cell–cell contact with breast carcinoma cells and bone marrow fibroblasts (Saad *et al*, 2000). Urokinase plasminogen activator is also found in human primary prostate carcinomas and in bone metastases (Kirchheimer *et al*, 1985). Data obtained from a limited number of cell lines have indicated that uPA is associated with the more aggressive type of carcinoma and facilitates invasion (Hoosein *et al*, 1991; Festuccia *et al*, 1998). These proteases are therefore of particular interest in the metastatic development of prostate cancer.

As yet, no human model has been established to study variations in expression and effects of MMP-1, MMP-7 and uPA on the formation of prostate bone metastases *in vitro*. The prostatic epithelial cell (PEC)/BMS co-culture system as described by Lang *et al* (1998) represents an ideal *in vitro* model for this purpose. Here, we propose that enzyme mediated cell interactions maybe important for the expansion of epithelial colonies within BMS.

## MATERIALS AND METHODS

### Materials

All reagents were purchased from Sigma-Aldridge, Poole, UK. All tissue culture medium and horse serum was from Life Technologies, Paisley, UK with the exception of Ham's F12 media, PAA Laboratories, Austria. Foetal calf serum (FCS) was supplied by Sera Labs, Sussex, UK and Worthington collagenase type 1 and trypsin from Lorne Laboratories Ltd., Twyford, UK.

### Antibodies

MMP-7 and uPA were from Chemicon International, Harrow, UK; MMP-1 and mouse IgG1 from R&D Systems, Abingdon, UK; mouse anti-human pan cytokeratin from Sigma-Aldridge, Poole, UK; rabbit anti-human pan cytokeratin from Biogenesis Ltd., Poole, UK; rabbit anti mouse biotinylated antibody, swine anti-rabbit TRITC and aqueous mounting medium from DAKO Ltd., Cambridge, UK. Vectastain Elite ABC kit from Vector Laboratories, CA, USA and streptavidin Alexa Fluor® 488 was from Molecular Probes, Oregon, USA.

### Cell lines

The malignant prostate cell line PC-3 (bone marrow metastasis derived) (Kaighn *et al*, 1979) and non-malignant line PNT2-C2 (Berthon *et al*, 1995) were cultured in Ham's F12, 7% FCS and 2 mM L-glutamine and in RPMI 1640, 10% FCS and 2 mM L-glutamine respectively. Cultures were grown at 37°C in a humidified atmosphere of 5% CO_2_ in air.

### Primary tissue collection and culture

With informed consent prostatic tissue was obtained from males undergoing treatment for malignant (CaP) or non-malignant benign prostatic hyperplasia (BPH) disease. Each individual prostate chip was bisected with half undergoing histological analysis for diagnostic evaluation and the remainder used for tissue culture. Prostate epithelia and fibroblasts were isolated by collagenase digestion followed by differential centrifugation as described (Lang *et al*, 1998). Epithelial cells were initially grown in flasks and then used at passage 1–3.

Human ribs removed for access during routine renal surgery were prepared for tissue culture using the method of Coutinho *et al* (1993). Bone marrow cells were flushed out from the rib and were resuspended in long-term culture medium (Iscove's Modified Dulbecco's Medium containing 10% FCS, 10% horse serum and 5×10^−7^ M hydrocortisone) and 2×10^7^ cells plated into 25 cm^2^ tissue culture flasks. The cultures were grown at 33°C in 5% CO_2_ in air for 4–5 weeks until haemopoietically active areas were observed.

### Co-culture experiments

Bone marrow stromal cultures were trypsinised (0.5% trypsin) and re-seeded into 24-well culture plates (or 4-well glass slide flask) at the same cell density and incubated for 48 h.

The media on the BMS was removed and replaced with long-term culture medium plus Keratinocyte-SFM (1 : 3) containing 500 epithelial cells (PC-3, PNT2-C2 or primary PEC) per well. The co-cultures were incubated for 7–10 days at 37°C then fixed with methanol/acetone (1 : 1), allowed to dry and frozen at −20°C until required.

### Immunocytochemistry

Samples were incubated with primary antibodies overnight at 4°C in a humidified chamber after blocking with 10% serum then 0.3% hydrogen peroxide. Anti-MMP-7, anti-uPA, anti-MMP-1 and mouse IgG1 were prepared at 20 μg ml^−1^ and mouse anti-human pan cytokeratin at 1 : 200. This was followed by addition of biotinylated rabbit anti mouse 1 : 400 for 40 min at room temperature. A complex of avidin DH and biotinylated horseradish peroxidase H (Vectastain Elite ABC kit) was then added for 15–20 min, followed by the addition of DAB substrate for 5–10 min. After washing with distilled water the cells were counterstained using haematoxylin and fixed in aqueous mounting fluid. Samples were scored as to the intensity of brown staining: (−) blue haematoxylin counterstain visible only, (−/+) pale brown, (+) brown, (++) dark brown. These staining intensities were set using agreed criteria and results were corroborated with more than one investigator.

For dual fluorescent staining, cultures were fixed, blocked and labelled for primary antibodies as observed above. Rabbit anti-human cytokeratin (1 : 100) was added for 30 min, followed by a swine anti-rabbit TRITC (1 : 20) for 1 h in the dark. The samples were blocked for 30 min using PBS/10% rabbit serum and 1% BSA, then incubated with rabbit anti-mouse biotinylated antibody (1 : 400) for 30 min. After washing, streptavidin Alexa Fluor® 488 (5 μg ml^−1^) was added for 1 h in the dark.

Cells were observed using an Olympus BX51 upright microscope, with a Plan-Apochromat 40× objective (NA 1.0, oil immersion). Using a triple-band bypass filter (Chroma Technology Corp. set: 61000 v2SBX for DAPI/FITC/TRITC) the interaction of TRITC/Cytokeratin (ex.542 nm em.560 nm), FITC/uPA, MMP-1, MMP-7 (ex.488 nm em.505–530 nm) and DAPI (ex.360 em.460) were examined. Images were captured via a cooled Colourview 12 and the analySIS imaging acquisition and processing system (SiS, GmbH). Images were captured at 1300×1030, 24 bit resolution with an exposure time of 5 s. Processing and montaging of images was then carried out using Adobe PhotoShop 6.0 (Adobe Systems Inc.)

### Immunohistochemistry

Prostatic bone marrow metastases were sectioned from 8 mm trephine core biopsies taken from the iliac crest of patients with untreated prostate cancer. The undecalcified paraffin embedded sections were first de-waxed in two changes of xylene for 5 min, then run through a series of alcohols to rehydrate the sections. Sections were immersed in citrate solution pH 6.0 and placed in a microwave on full power for 25 min. The solution was allowed to cool and was exchanged for water. Samples were stained and scored according to protocols shown above.

### Inhibition studies

Primary epithelial cell/BMS co-cultures were set up as above, in addition to which the epithelial cells were initially allowed to bind to the BMS for 4 h at 37°C before antibodies against either uPA, MMP-1 and MMP-7 (functionally blocking) were added at 2 μg well^−1^ daily. Mouse IgG1 was added to the control well. After 7 days at 37°C the cultures were fixed using methanol/acetone (1 : 1), dried then immunostained for cytokeratin. Colony areas were measured using an eyepiece graticule after calibration with a slide graticule. For each well >100 colonies were measured for length and width. The median colony area per well was then calculated and statistical analysis achieved using Wilcoxon's Rank Sum Test.

## RESULTS

### Epithelial colony morphology and enzyme characterisation in BMS co-culture

**Figure 1:**
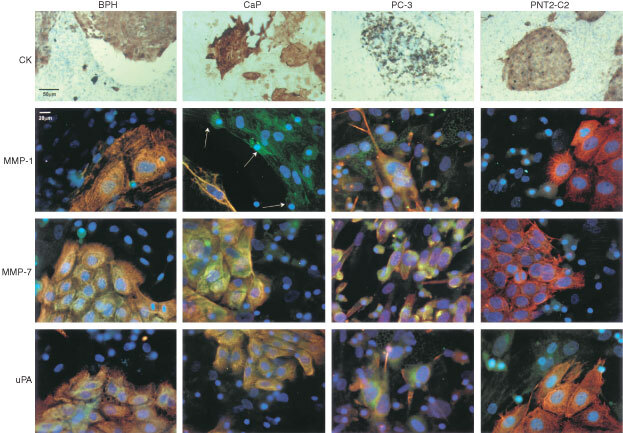
Enzyme characterisation of primary prostate epithelial cells and the prostate cell lines grown with BMS. Immunocytochemical staining showing typical colonies of cytokeratin (CK) positive (DAB brown staining) and fluorescent dual staining of epithelial cells grown in BMS co-culture (each picture is unrelated). Enzyme stained photographs are shown as dual fluorescence: red=cytokeratin; green=location of either uPA, MMP-1 or MMP-7; yellow=double staining; blue=DAPI nuclear counterstain (*n*=5). All negative controls were black. Arrows indicate retracted stromal edge.

The cell lines PC-3, PNT2-C2 (derived from normal prostate), and primary epithelial cells (derived from patients with CaP or BPH) were initially grown on BMS and stained for cytokeratin to observe colony morphologies. As a comparison to the malignant cell line PC-3, the PNT2-C2 cell line was used to represent normal prostate cell behaviour. We also used primary PEC derived from patients with CaP and compared them to BPH derived epithelial cells. Figure 1

(CK) shows the appearances of the different epithelial cells grown on BMS and labelled with cytokeratin. Primary epithelial cells derived from patients with CaP grew colonies that appeared either spiky or round and compact. Epithelial cells derived from patients with BPH always formed round and compact colonies. Retraction of the BMS was also seen with cells derived from CaP. Cell lines showed dispersed PC-3 colonies but compact PNT2-C2 colonies.

Co-cultures were also dual-stained for cytokeratin, and either MMP-1, MMP-7 or uPA to establish that prostate epithelial colonies produce these enzymes in BMS. Epithelial cells derived from CaP stained positively for MMP-1 and MMP-7 when grown in BMS co-culture (Figure 1). Some of the bone marrow stroma at the interface with the PEC (orange/yellow) had retracted away and showed intense MMP-1 green staining (arrows). Urokinase plasminogen activator staining was positive but less intense. Colonies from BPH derived epithelial cells showed staining of MMP-1, MMP-7 and uPA located within the PEC with less marked staining towards the edge of the colony.

Co-cultures of the cell lines showed that all three enzymes were observed throughout the PC-3 cells. This co-localised with cytokeratin at the cell membrane/pseudopodial processes. The PNT2-C2 cell line showed some weak cytoplasmic staining for all the enzymes, but they were predominantly cytokeratin positive.

To compare the presence of MMP-1, MMP-7 or uPA when epithelial cells (cell lines and primaries) were grown on tissue culture plastic or normal human BMS, cultures were immuno stained, using DAB. The results summarised in Table 1

**Table 1:**
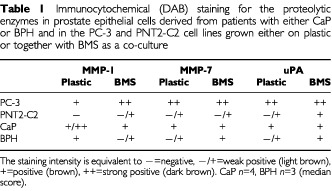
Immunocytochemical (DAB) staining for the proteolytic enzymes in prostate epithelial cells derived from patients with either CaP or BPH and in the PC-3 and PNT2-C2 cell lines grown either on plastic or together with BMS as a co-culture

showed that the PC-3 cell line expressed strong staining for all the enzymes tested on plastic, except MMP-1, but this was up-regulated in the presence of BMS. The non-malignant cell line PNT2-C2 demonstrated absent or low staining intensities for all the enzymes on plastic but showed up-regulation of MMP-1 and uPA on BMS, with uPA staining the most. Primary PEC cultures from patients with CaP showed positive staining for all the enzymes on plastic, which was more intense than in the cells derived from BPH. There was no difference in staining between CaP and BPH on BMS for MMP-7 and uPA. However, MMP-1 expression was greater in CaP derived cells compared to BPH. These results were consistent in several primary and cell line cultures.

### Enzyme characterisation of bone marrow metastases

**Figure 2:**
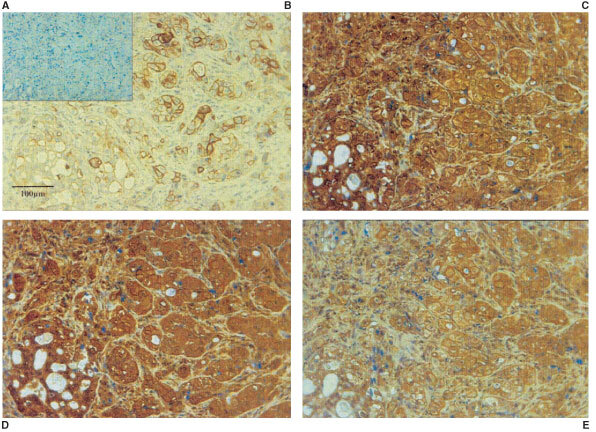
Serial sections of a prostatic bone marrow metastasis. Typical examples of the enzyme staining found from seven samples (see Table 2). Each photograph shows the same region of sample immunohistochemically (DAB) labelled for: (**A**) negative control (haematoxylin), (**B**) cytokeratin indicating the colonies of epithelial cells within the bone marrow space, (**C**) MMP-1, (**D**) MMP-7 and (**E**) uPA.

Using bone marrow metastasis sections from patients with CaP it was possible to compare the staining pattern of the enzymes *in vivo* to the *in vitro* model. These sections were previously identified as prostatic bone marrow metastases by positive PSA staining (Bryden *et al*, 2002). Figure 2

shows immunohistochemical (DAB) staining for MMP-1, MMP-7 and uPA using the same concentration of antibody as for the co-culture staining. Serial sectioning allowed comparison of the different enzyme staining patterns within the same area of tissue. Areas of cytokeratin positive epithelial cells within the bone marrow structure can be seen in Figure 2B. The epithelial colonies showed high expression of MMP-1 (C) and MMP-7 (D), with less intense staining in the surrounding stroma. Figure 2E showed weaker staining for uPA than was observed for MMP staining, but again this was more clearly stained in the epithelial region than the stromal area.

Prostate bone metastases (*n*=7) were sectioned and stained for cytokeratin, MMP-1, MMP-7 and uPA. Median staining scores were then calculated and summarised in Table 2

**Table 2:**
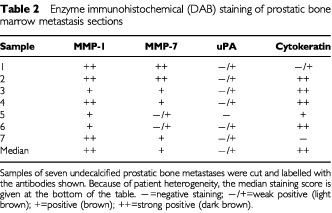
Enzyme immunohistochemical (DAB) staining of prostatic bone marrow metastasis sections

. One sample was cytokeratin negative but was identical in morphology to the cytokeratin positive epithelia in the other samples. Generally samples stained strongest for MMP-1, then MMP-7 and more weakly for uPA. The staining intensity was strongest towards the outer edges of the epithelial cells.

### Inhibition studies

**Figure 3:**
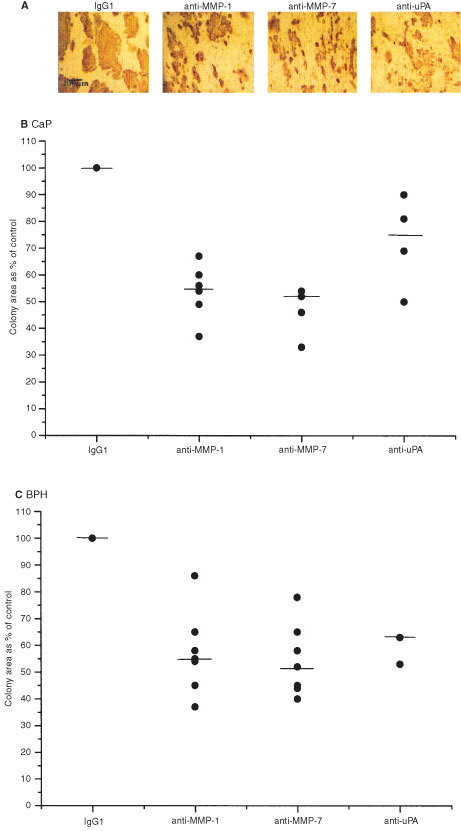
Antibody inhibition of epithelial colony area (%). A comparison of the inhibition of prostate epithelial colony area in the presence of enzyme antibodies was made for a variety of primary epithelial samples. Typical immunocytochemical (DAB) cytokeratin staining of a BPH epithelial cell/BMS co-culture in the presence of non-specific mouse IgG1, anti-MMP-1, anti-MMP-7 and anti-uPA antibodies (2 μg day^−1^) after 7 days in culture is shown in (**A**). Median area of >100 colonies per sample were calculated and inhibition was calculated as a per cent of the control area. Median percentage inhibition is represented by the bar: (**B**) CaP, MMP-1 and MMP-7 (*n*=6), uPA *n*=4; (**C**) BPH, MMP-1 and MMP-7 (*n*=7), uPA (*n*=3).

Functionally blocking antibodies against the proteolytic enzymes were added to the *in vitro* co-culture system to further study the role of these enzymes in epithelial colony formation. As the enzymes are secreted continually by the epithelial or stromal cells, antibodies were added daily. Titration of the antibodies indicated that 2 μg ml^−1^ consistently and significantly inhibited growth of epithelial colonies (data not shown). Figure 3A

shows the appearance of typical BPH derived colonies after treatment with the antibodies, cultures with samples derived from patients with CaP showed a similar appearance. The control co-culture showed large colonies, whereas the co-cultures treated with the blocking antibodies showed smaller irregularly shaped colonies. Results were similar whether epithelial cells were derived from benign or malignant tissue. This experiment was repeated on primary epithelial cell samples, derived from malignant (*n*=6) or benign tissue (*n*=7) shown in Figure 3B,C. Patient samples varied in response to these blocking antibodies, but overall, samples derived from patients with CaP and BPH showed greatest reduction in colony area with anti-MMP-7, followed by anti-MMP-1 then anti-uPA. Median per cent reduction of colony area for CaP derived colonies compared to the control was 48, 45 and 23%, with BPH derived colonies 48, 45 and 37% respectively. There was no significant difference observed between the responses for CaP or BPH derived epithelial cells. However, statistical analysis showed significantly reduced (*P*<0.05) colony growth after anti-MMP-1 was added to the culture in 6/6 CaP samples and 6/7 BPH samples. Inhibiting MMP-7 significantly decreased (*P*<0.05) colony size in 5/6 CaP and 7/7 BPH samples and blocking uPA activity significantly reduced (*P*<0.05) growth in 1/3 CaP and 4/4 BPH samples (Wilcoxon's Rank Sum Test) compared to the control.

The total numbers of colonies formed in the presence of the antibodies was variable, either remaining the same or increasing above that of the control (data not shown).

## DISCUSSION

Prostate cancer shows a predilection to metastasise to the bone marrow. We have used *in vitro* models (Lang *et al*, 1998) to identify potential enzymes involved in this process and compared these with *in vivo* effects in human bone marrow metastasis sections.

Initially we looked at the colony morphology of PEC when grown in BMS. The PC-3 cell line and cells derived from patients with CaP showed colonies and cells which appeared spiky and scattered whilst the PNT2-C2 cell line and cells derived from patients with BPH showed rounded colonies. This confirmed findings previously reported (Lang *et al*, 2000) which suggests a more motile and invasive nature of PC-3 and CaP derived epithelial cells. Regarding proteolytic enzyme production in BMS co-culture, we found that when cells derived from patients with CaP were grown in BMS co-culture, not only were the epithelial cells strongly stained for MMP-1, the stromal cells around the leading edge of the colony were also strongly positive. This has been shown in previous studies where *in vivo* MMP-1 expression was found in the stromal area along the invasive margin in gastric cancer (Migita *et al*, 1999) and breast cancer (Heppner *et al*, 1996). Also, *in vitro* MMP-1 expression was up-regulated by cell–cell contact between BMS and breast carcinoma cells (Saad *et al*, 2000). This may indicate subtle tumour/stroma interactions causing increased secretion of these enzymes. Epithelial cells derived from patients with BPH showed enzyme staining similar to CaP derived cells but less was seen at the stromal margins of these colonies, suggesting no stimulation of the stromal cells to secrete these enzymes. Clearly interactions between BMS and epithelial cells play an important role in the level of enzyme expression.

Using immunocytochemistry we compared the enzyme levels between the cell lines PC-3, PNT2-C2 and the primary PEC. Previous studies showed that the PC-3 cell line expresses uPA (Hoosein *et al*, 1991) and mRNA for 10 MMPs including MMP-1 and MMP-7 (Giambernardi *et al*, 1998). We confirmed that the PC-3 cell line gave strong positive staining for uPA, MMP-1 and MMP-7. By comparison, the PNT2-C2 cell line gave weak staining for these enzymes, which was marginally increased in BMS co-cultures. For the primary PEC, median staining values were shown due to patient variability. This phenomenon must not be overlooked as differing enzyme expression between patients could provide an insight into why prostate cancer can be highly variable in its disease progression. Generally, primary samples from patients with CaP stained more strongly for these enzymes than samples derived from BPH when grown on tissue culture plastic. However, apart from MMP-1 (increased staining for CaP in BMS compared to BPH cells) we did not find a difference between the staining of CaP or BPH derived samples when grown in the BMS co-culture. In support of this, it has been reported that both CaP and BPH epithelial cells will grow and invade in BMS co-culture in a similar way (Lang *et al*, 1998), despite the fact that although BPH cells do not invade the bone marrow *in vivo* they are released into the circulation during routine TURP for bladder outflow obstruction (McIntyre *et al*, 2000, 2001). Therefore, subtle differences in enzyme expression between CaP and BPH epithelial cells could make a difference to these circulating cells in determining their ability to invade through endothelium and ultimately grow in the bone marrow itself.

Our results indicated very subtle differences in enzyme expression between the CaP and BPH primary epithelial cells when grown in BMS whereas in previous *in vivo* studies greater differences were observed. Hashimoto *et al* (1998) demonstrated a significant correlation between the levels of MMP-7 mRNA in the primary prostate carcinoma and pathological stage and mean serum levels of uPA were reported to be significantly higher in patients with CaP compared to healthy individuals or patients with BPH (Miyake *et al*, 1999). However, no significant correlation was made in a study carried out to identify changes in blood plasma levels of MMP-1 between patients with CaP or with BPH (Lein *et al*, 1998).

Our *in vivo* staining of prostate bone marrow metastases from patients with CaP showed positive staining for MMP-1 and MMP-7 but less intense staining for uPA. Previous work has demonstrated that MMP-1 levels in uncultured primary prostate carcinoma are very low and variable (Varani *et al*, 2001), whereas we have demonstrated that MMP-1 is strongly expressed in the bone metastases of prostate cancer patients. Our *in vitro* data also showed that in BMS co-culture, MMP-1 staining was greater in CaP co-cultures compared to BPH co-cultures. This reiterates the proposition that up-regulation of MMP-1 secretion maybe an important factor in the formation of prostate bone marrow metastases. As these changes in enzyme expression were subtle, further work using zymography may clarify these results and give quantitative data to corroborate our qualitative data.

Until now there has been no human *in vitro* model to demonstrate the role of proteolytic enzymes on metastasis formation, and there have been few studies to determine whether blocking these enzymes causes significant effect (although, previous animal models have shown that rats inoculated with Dunning R 3227, Mat LyLu rat prostate carcinoma cell line transfected to over-express uPA developed hind limb paralysis much faster than transfectants which under-expressed uPA (Achbarou *et al*, 1994)).

The BMS co-culture system was used as a human ex-vivo model to study the effects of blocking enzyme activity on prostate epithelial colony growth enabling direct observation of their importance in the process of invasion. In most samples total median colony area was reduced by around 50% following inhibition of MMP-1 and MMP-7. For uPA the reduction was less but still significant. In this study, cells from the primary tumour site rather than cells from metastatic bone biopsies were grown. Kirchheimer *et al* (1985) showed that out of 14 primary prostatic carcinomas and their respective bone metastases, the bone metastases had 1.5 times higher uPA than the corresponding primary tumour. For future study it would be interesting to ascertain whether greater reduction in colony size could be achieved by using prostate bone metastatic cells in the BMS co-culture.

Blocking MMP enzyme function caused reduction in colony size but not their number. Therefore, initial establishment of epithelial colonies within BMS was not affected although the subsequent growth and formation of these colonies was. Recent evidence has revealed that the MMPs not only cause degradation of the ECM but also interact with adhesion molecules and can stimulate cell migration and invasion. What we observed may have been therefore the result of cell migration and colony formation failure due to the neutralising effect of the antibodies (Stetler-Stevenson and Yu, 2001).

In summary, we have used the *in vitro* BMS co-culture system to show that MMP-1, MMP-7 and uPA are expressed by the bone metastatic prostate cell line PC-3, primary CaP and BPH derived epithelial cells. They are also up-regulated in the non-malignant prostate cell line PNT2-C2 when grown within BMS. We have demonstrated similar staining results using human prostate bone marrow metastases sections *in vivo* confirming the notion that *in vivo* observations reflect events observed *in vitro*. Inhibiting these enzymes reduced prostatic colony growth in the BMS, a finding supporting the proposition that these enzymes are important in development of metastases in humans. Our understanding of the metastatic process regarding MMP involvement is still limited, but clearly these enzymes play an important role in the invasion of bone marrow stroma by prostatic epithelial cells and bone marrow metastasis formation in man.
